# MOLECULAR DETECTION PROTOCOL OF SARS-COV-2 THROUGH SELF-COLLECTED SALIVA SPECIMENS VERSUS NASOPHARYNGEAL SWABS

**DOI:** 10.21010/Ajidv18i2.1

**Published:** 2024-03-08

**Authors:** GHIDOUCHE Abderezak, HALLOUCHE Sarah, AIT-ALI Djida, BOUDRAHEM-HANNOU Lila, NOURI Hamid, TLIBA Souhil, BITAM Idir, AMIROUCHE Adel

**Affiliations:** 1 Faculté des Sciences de la Nature et de la Vie, Université de Bejaia, Bejaia, Algérie; 2 Laboratoire de Génie Biologique des Cancers, Université de Bejaia, Bejaia, Algérie; 3 Faculté de Médecine, Université de Bejaia, Bejaia, Algérie; 4 Service des maladies infectieuses, CHU de Bejaia, Algérie; 5 Laboratoire de Microbiologie Appliquée, Université de Bejaia, Bejaia, Algérie; 6 Service de Neurochirurgie, CHU de Blida, Algérie; 7 Ecole Supérieure en Sciences de l’Aliment et des Industries Agroalimentaires (ESSAIA), El Harrach, Alger, Algérie; 8 Aix Marseille Univ, IRD, VITROME, IHU Méditerranée Infection, Marseille, France

**Keywords:** Diagnosis, Nasopharyngeal swab, Reverse Transcriptase Polymerase Chain Reaction, Saliva, SARS-CoV-2

## Abstract

**Background:**

Several reports have shown that saliva specimen is an excellent alternative biofluid sample for SARS-CoV-2 detection. We conducted this study, in order to assess the sensitivity and specificity of using saliva self-collected by adult and pediatric patients, as a biological sample for RT-PCR diagnosis.

**Aims:**

The present study was carried out to assess the sensitivity and specificity of using saliva self-collected from adult and pediatric patients, as a biological sample for RT-qPCR diagnosis.

**Methods:**

In this study, 50 symptomatic patients and 40 asymptomatic subjects (adult and pediatric) were enrolled between September 2020 and November 2020 at the Department of Infectious Diseases, Bejaia University Hospital (CHU), and tested simultaneously for the sensitivity and specificity of the SARS-CoV-2 viral genome by RT-PCR on both nasopharyngeal swabs NP swab and saliva samples.

**Results:**

Our RT-qPCR results revealed that saliva samples showed the highest sensitivity (95% CI [27.67, 29.82]) followed by a nasopharyngeal swab for symptomatic (95% CI [29.64, 31.49]) as well as for asymptomatic adult patients. Moreover, the saliva of symptomatic and asymptomatic patients was monitored, and the presence of viral RNA was detected in >95% of the asymptomatic patients as well as the symptomatic patients. Surprisingly, the Ct values of ORF1ab and N genes are highly lower in nasopharyngeal swabs compared to saliva. Indeed, the mean difference note that for the ORF1ab gene and N gene, the mean of difference in ΔCt value were respectively 3.683 and 3.578. Together, including symptomatic and asymptomatic subjects, the overall agreement between the saliva sample and the nasopharyngeal is about 84%.

**Conclusion:**

The sensitivity of saliva samples remains acceptable; it may still be a viable option in locations where laboratory facilities are lacking for diagnostic purposes in the early phase of the disease.

## Introduction

Severe Acute Respiratory Syndrome Coronavirus-2 (SARS-CoV-2) was reported for the first time in Hubei province (China). Since the end of 2019, Covid-19 became a global pandemic with more than 551 million cases diagnosed and 6.43 million deaths recorded to date. SARS-CoV-2 is a pathogenic member of the Coronavirinea subfamily and Betacoronavirus genera. It is an enveloped, non-segmented, single-stranded, positive sense RNA of ~29.9 kb (Mousavizadeh and Ghasemi, 2021). A typical representation of Coronavirus (CoV) contains relatively six Open Reading Frames (ORFs) in its genome. The first ORFs (ORF1a/b), which account for about two-thirds of the total genome length. Four major structural proteins containing the envelope (E), membrane (M), nucleocapsid, (N) proteins and protein S (Spike) and are encoded by ORFs 10, 11 near the 3-terminus (Van Boheemen *et al.*, 2012). SARS-CoV-2 binds to angiotensin-converting enzyme 2 (ACE2), is highly expressed in the lungs, heart and kidneys, through its spike protein and thus allows SARS-CoV-2 to enter the host cells and causing subsequent pathogenicity (Beyerstedt *et al.*, 2021). In order for the virus to complete entry into the cell following this initial process, the spike protein has to be primed by an enzyme called a protease.

Similar to SARS-CoV-1, SARS-CoV-2 uses a protease called Transmembrane Serine Protease 2 (TMPRSS2) to complete this process (Báez-Santos *et al.*, 2015). Activation by TMPRSS2 as a protease is needed to attach virus receptor (spike protein) to its cellular ligand (ACE2) (Hoffmann *et al.*, 2020).

From November 24, 2021, numerous variants of SARS-CoV-2 have begun to attract public health interest, including variant alpha (B.1.1.7), variant beta (B.1.351), variant gamma (variant P.1), (Laiton-Donato *et al*. 2021), variant delta (B.1.617.2)(Raman *et al.*, 2021), variant Lambda (C. 37) and most recently variant Omicron. It was first discovered in specimens collected on November 11, 2021 in Botswana and on November 14, 2021 in South Africa, it gained attention from its spread (Karim and Karim 2021). Like the variant delta, the Omicron variant, harboring multiple mutations in the spike protein, gives it marked resistance compared to other strains of SARS-CoV-2, such as the alpha strain (Karim and Karim 2021; Scott *et al*. 2021). Mutations in the spike protein decrease the efficacy of many of the currently available vaccine options and present an ongoing challenge to the SARS-CoV-2 pharmacological strategy (Mohammadi *et al.*, 2021).

The effectiveness of the RT-PCR screening strategy depends on the number of tests performed, but also the time taken to report the results. Viral nucleic acid extraction using an RNA kit is the gold standard for diagnosis of SARS-CoV-2 infection by quantitative reverse transcription polymerase chain reaction (RT-qPCR). One of the limiting factors involved in the lengthening of the time is due to the nasopharyngeal swab sampling step. In response to the high demand for screening and diagnostic tests, efforts have been made to develop a simplified protocol for the isolation of SARS-CoV-2 viral RNA.

Due to the shortage of authorized health workers, their high risk of Sars-Cov2 infection while carrying out the sampling, but also the invasive and unpleasant nature of the act of sampling, many strategies are being studied in order to substitute RT-PCR screening with other techniques or use another type of biological sample that is easier to collect, such as saliva. Several reports have shown that saliva specimen is an excellent alternative biofluid sample for SARS-CoV-2 detection. The advantages of the saliva testing include the noninvasiveness, the easier sample collection in particular from fragile patient populations and reduction in the potential risk of infection. We conducted this study, in order to assess the sensitivity and specificity of using saliva self-collected by adult and pediatric patients, as a biological sample for RT-PCR diagnosis.

## Materials and Methods

### Study Design and Participants

In this observational study, a total of 75 adult patients were enrolled between September 2020 and November 2020. The patients were included in this study after providing informed consent. In addition to adult subjects, we included young subjects (n=15, age ≤ 12 years) in the study. All samples collected were anonymized using an alpha- numeric identification code, and the study was approved by the Local Ethics Committee.

### Nasopharyngeal and Saliva Sample Collection

Symptomatic or asymptomatic patients were examined for SARS-CoV-2 by RT-qPCR using nasopharyngeal swabs and saliva specimens collected at the Department of the Infectious Diseases, Bejaia University Hospital (CHU), in accordance with the nationally recommended method in Algeria. Depending on the presence or absence of symptoms, the patients were divided into two groups, symptomatic patients (n = 50) and asymptomatic patients (n = 25). Asymptomatic patients are subject-contact of symptomatic people or family cluster.

A nasopharyngeal swab and a saliva collection were taken from each the patients. NP specimens were collected by midturbinate swabbing of both noses and the posterior pharynx, avoiding the tongue. For saliva collection, a non- invasive method was executed by making self-collected specimen in a tube container. Only saliva sample is taken for each child.

The samples were obtained with nasopharyngeal swabs or saliva collection during the clinical course at the Department of Infectious Diseases, Bejaia University Hospital. A sterile tube was provided for the patients, and they were requested to self-sample (500 μl). After cessation of eating or drinking for at least 30 minutes, the oral cavity of each patient was cleaned before saliva collection.

However, some rules must be followed before carrying out the self-sampling. Saliva samples containing salivary mucus are excluded from the study. The salivary samples are vortexed for 15 seconds and stored at 4 °C overnight.

### Viral load monitoring

For viral load monitoring, three patients were asked to produce an early morning saliva sample and we decided to assess the saliva for SARS-CoV-2 positivity by using RT-qPCR technique. Ct values of two molecular targets, Orf1ab and N genes, were monitored at day 1, 2, 4, 6 and 8 after SARS-CoV-2 detection from the saliva from three patients.

### RNA extraction:

The extraction of viral RNA from the different samples (Nasopharyngeal & Saliva) was carried out by an automatic extraction system using the Nuactor^®^ Viral RNA Extraction Kit (Boditech Med Incorporated). Extraction of viral RNA using the Nuactor^®^ system is based on the use of a cartridge containing magnetic beads, elution solutions and lysis solution. According to the manufacturer’s recommendations, 200μl of sample was added to 700μl of lysis buffer previously placed in the cartridge. The cartridge was then placed in the machine and the viral RNA extraction process was carried out.

### Real-time PCR

A real-time quantitative PCR was performed on StepOnePlus real-time PCR system (Applied Biosystems^®^) using a detection Novel coronavirus (2019-nCoV) Nucleic Acid Detection Kit (PCR Fluorescence Probing) (Shangai BioGerm Medical Technology Co., Ltd). This kit is based on one-step RT-PCR technique with the following cycle parameters: 10 minutes at 50 °C for reverse transcription, 5 minutes inactivation at 95 °C followed by 40 cycles of 10 seconds at 95 °C and 40 seconds at 55 °C. ORF1ab and N regions are highly conserved amongst sarbecoviruses, these were selected for probe designs and primer. Specific primers and fluorescent probes are designed (ORF1ab gene probe is labeled with FAM and N probe with VIC) for the detection of 2019 novel coronavirus RNA in specimens.

When the Ct value is equal or greater than 38, the specimen is considered negative. Internal control used in the test is the RNAse P gene; the probe for internal control is labelled with ROX (Amirouche *et al*. 2021).

### Statistics

Various statistical tests including paired and unpaired t- tests, were used to determine whether specific group mean differences were significant. Linear regression analysis was used to assess the relationship between each molecular diagnostic method. For reliability or precision, Cohen’s Kappa coefficient was used. Each performed test is specified in the figure legends. The minimum α-level of significance was set at 0.05. Data are presented as means+ SEM throughout.

## Results

Main characteristics of the patients from nasopharyngeal and saliva samples. Effect of clinical background against the presence of viral RNA.

Based on previous reports showing that saliva is easily collected and recognized as promising biological matrix for early detection of respiratory infections, we examined the effect of clinical background against the presence of viral RNA.

In this study, 31 patients (66%) were classified SARS-CoV2 symptomatic and 08 patients (33%) had no symptoms of SARS-CoV2 at the time of testing. InSARS-CoV-2 symptomatic patients there were 15 males, and 16 female patients (Table 1). Patient age ranged from 26 to 87 years, the median age was 58.28 ± 14.4 years for symptomatic patients and 44.23 ± 17 years for asymptomatic patients (Table 1). It should be noted that the median age was 7.75 ± 2.5 years for pediatric patients (Table 1).

**Table 1:** Main characteristics of the patients according to the RT-qPCR result from nasopharyngeal and saliva samples. Note that we performed two RNA extractions for each patient.

	Age Median(±SD)	Gender		Nbre of NPS positive	Nbre of Saliva positive
		% of Male	% of Female		
Symptomatic (n=50)	58.28 (±14.48)	35.4	64.6	36	28
Asymptomatic (n=25)	44.23 (±17.01)	48	52	00	00
Children (n=15)	7.75 (±2.517)	64.28	35.72	/	00

### Quality of sampling according to RnaseP gene Cycle threshold value by RT-PCR.

**Figure 1:**
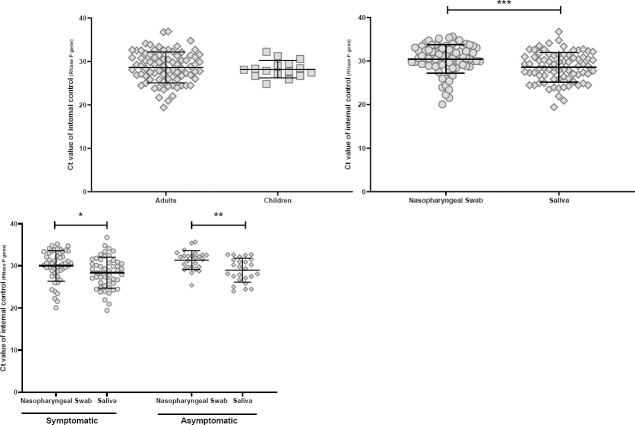
Quality of sampling according to Rnase P gene Cycle threshold value by RT-PCR. A: Comparison between Ct value of Rnase P for adult subjects (n=75) and children group (n=15). B: Comparison between Rnase P Ct values of Nasopharyngeal swab (n=75) and Saliva from adult subjects (n=75)***: *p-value=*0.0003. C: Comparison between Rnase P Ct values of Nasopharyngeal swab (n=75) and Saliva from symptomatic adult subjects (n=50) (*p-value* :0.0173) and Asymptomatic subjects (n=25) (*p-value* :0.0054). Noted that, we performed two RNA extraction for each patient. Error bars denote mean ± standard error of the mean (SEM) for two technical replicates. *P* values were determined by using paired and unpaired t- tests.

Collectively (symptomatic with asymptomatic patients), we have shown that Ct values of RNAseP, used as internal control-levels, are significantly (P<0.001) lower in saliva (Ct=28.566 ± 3.428) compared with nasopharyngeal samples (Ct = 30.453 ± 3.28) (Figure1A). Significant difference (P<0.05) was also found in symptomatic patients when we compared saliva specimens (Ct = 28.75 ± 3.87) with conventional NPS (Ct = 30.57 ± 3.35) (Figure1B). This effect was not only observed in symptomatic patients but also, and importantly (P<0.01), in asymptomatic patients (Saliva: Ct = 28.56 ± 3.09 and NPS: Ct = 30.344±2.715) (P<0.001) (Figure 1B). It is noteworthy that there were no significant differences in detection rates of RNA viral of saliva of symptomatic patients compared with asymptomatic patients. On the other hand, we examined whether there was a difference of Ct values of RNaseP between adult specimens’ saliva and paediatric saliva (Figure 1C). RNaseP Ct values of saliva samples recruited from pediatric patients (Ct = 28.216 ± 1.958) are similar in saliva specimens (Ct = 27.97) compared to adult saliva specimens (Ct = 28.566 ± 3.428). Together, these data demonstrate that the detection rate of viral RNA in saliva was significantly higher than conventional NPS and independently of clinical background. Moreover, the saliva of symptomatic and asymptomatic patients was monitored, and the presence of viral RNA was detected in >95% of the asymptomatic patients as well as the symptomatic patients. Here, 48% represent 36 cases of 75 patients were tested positive for nasopharyngeal nucleic acid detection. For all the adult patients enrolled in the study, 48% (36/75) tested positive for SARS-CoV2 by using nasopharyngeal swab and 28/75positive samples (37.33%) had SARS-CoV-2 detected in saliva. However, 26 of patients had positive PCR in both samples NPS and saliva (34.66%) (Table 2).

**Table 2:** Comparison of sensitivity and specificity according to Ct value of internal control gene (Rnase P) of the saliva samples as compared with the result of nasopharyngeal samples.

	Ct Value RNase P from NPS	Ct Value RNase P from Saliva
Symptomatic Adult (n=50)	30.57±3.35	28.75±3.87
	95% CI [29.641,31.499]	95%CI[27.677,29.823]
Asymptomatic Adult (n=25)	30.344±2.715	28.56±3.09
	95% CI[29.28,31.408]	95% CI[27.349,29.771]
Adult (n=75)	30.453±3.28	28.566±3.428
	95% CI[29.711,31.195]	95% CI[27.79,29.342]
Children (n=15)	/	28.216±1.958 95% CI[27.225,29.207]

CI : confidence interval calculated with exact method

NPS : nasopharyngeal swab

Only two patients who were monitored positive SARS-CoV-2 salivary results and their nasopharyngeal swabs were negative. The median cycle threshold (Ct) value was significantly lower in saliva compared with NPS in symptomatic patients as well as in asymptomatic patients (Figure 1C). This suggested that a viral load is higher in saliva compared with NPS, which may reflect differing quality between saliva and NPS collection.

**Figure 2:**
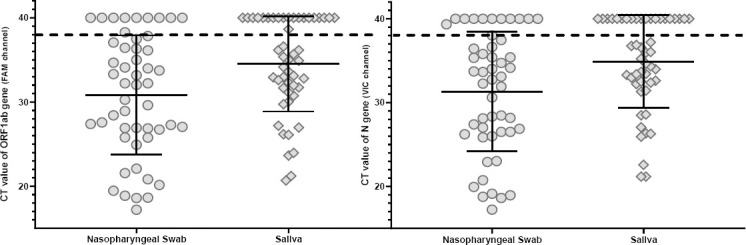
Cycle threshold value of SARS-Cov2 target genes (ORF1ab and N gene) in adults NPS compared with saliva samples. A: Cycle threshold value distribution of Orf1ab gene for Nasophargeal Swab (n= 50) vs Saliva Symptomatic adult (n=50) B: Cycle threshold value distribution of N gene for Nasophargeal Swab & Saliva of Symptomat adult (n=50). The dotted line represents the Ct positivity threshold according to the manufacturer of RT-qPCR SARS-CoV-2 detection kit. Noted that, we performed two RNA extraction for each patient. Error bars denote mean ± standard error of the mean (SEM) for two technical replicates. *P* values were determined by using paired t- test.

Comparison of sensitivity and specificity of target genes in saliva samples compared with the result of nasopharyngeal samples. Based on these observations, we then proceeded to determine whether this effect was also observed for ORFIab and for N gene. Detection of both targets and/or ORF1ab alone defines viral presence, while detection of N alone is considered a presumptive positive result (Amirouche *et al*. 2021). Based on these observations, we then proceeded to determine whether this effect was also observed for ORFIab and for N gene. We compared the Ct values of both targets ORF1ab and N gene. Surprisingly, the Ct values of ORF1ab and N genes are highly lower in nasopharyngeal swabs compared to saliva (Figure 2A and 2B). Indeed, we note that for the ORF1ab gene the mean of difference in Ct value is 3.683. For the N gene, the mean difference in Ct value is comparable to that observed for the ORF1ab gene (ΔCt = 3.578) (Figure 2A and 2B).

In addition, we observe that the Ct values of ORF1ab gene for nasopharyngeal swabs and saliva were significantly paired (Correlation coefficient (r) :05921, *p-value :<0,0001*). For the second target, N gene, a significant correlation was also observed between Ct-values for nasopharyngeal swabs and saliva (Correlation coefficient (r) :05610, *p-value :<0,0001*)).

### Effect of temporal profile of serial viral from saliva

**Figure 3:**
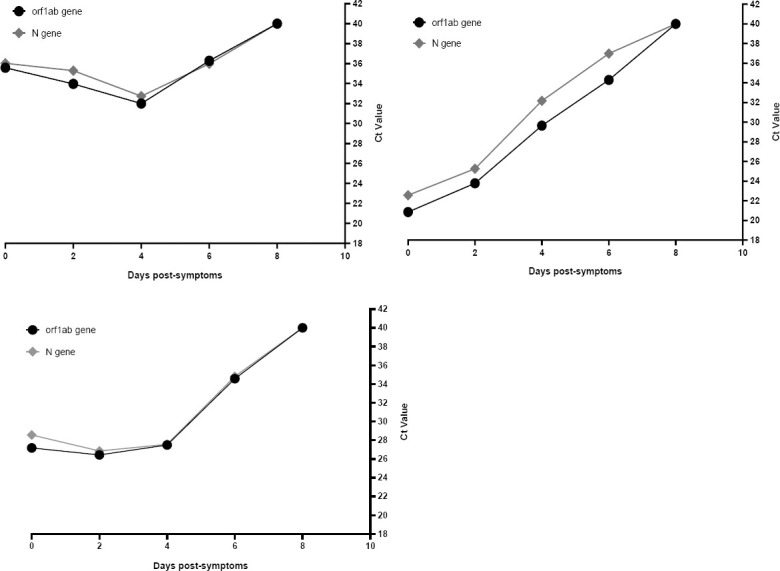
Evolution of target genes Cycle threshold values in saliva samples, according to time. The monitoring of Ct values of Orf1ab & N genes for SARS-CoV-2 is carried out by sampling patients saliva at an interval of two days. D_0_ represents the first sampling, A: 37-year-old male patient is. B: 67-year-old female patient. C: 42-year-old female patient. Noted that, we performed two RNA extraction for each patient.

In the most studies of respiratory virus infections, the serial viral sampling of nasopharyngeal or throat swabs was used for monitoring the clinical progress (K. K.-W. To *et al*. 2020; K. K. To *et al*. 2017). In the present study, we decided to assess the duration of saliva SARS-CoV-2 positivity by using RT-qPCR technique, Ct values of two molecular targets, Orf1ab and N gene, were monitored at day 1, 2, 4, 6 and 8 after SARS-CoV-2 detection from the saliva from three patients. Noted that saliva specimens’ collections were obtained at the same time. The decline of Ct value was progressive and reached at day 8 for both molecular targetsOrf1ab and N gene (Figure 3A, 3B and 3C).

Moreover, for all patients, the length of time required for samples to turn negative is 8 days onset SARS-CoV-2 detection. In addition, we have observed that saliva remains positive during six (06) days onset the first positive SARS-Cov-2 detection. Finally, including symptomatic and asymptomatic subjects, with samples kept overnight at 4°C and no prior treatment of the saliva samples -conditions which most closely resemble a systematic screening procedure, the overall agreement between the saliva sample and the nasopharyngeal is of the order of 84% and Cohen’s Kappa coefficient is estimated to 0.6767.

## Discussion

In this study, a total of 26 individuals with SARS-CoV2 were included and the assay was validated for both saliva samples and nasopharyngeal swab (NPS). Our proposed approach investigates the sensitivity and specificity of using saliva self-collected from adult and pediatric individuals, as a biological sample for RT-PCR diagnosis, to enable rapid and widespread SARS-CoV-2 testing.

We demonstrated higher sensitivity in detecting SARS-CoV-2 in nasopharyngeal swab compared to saliva samples. The vast majority of research and development today are designed for nasopharyngeal samples(Jamal *et al*. 2021; Butler-Laporte *et al*. 2021). However, using saliva as a substitute of nasopharyngeal swabs has numerous advantages. It is a non-invasive technique, easy to self-collect with little discomfort, risk of viral transmission during testing, does not require specialized health care personnel (Tsang *et al*. 2021).

NPS and other clinical specimens may provide higher sensitivity for viral detection. Today, there are few research and development designed for saliva available that have been thoroughly validated. Several studies have evaluated the use of saliva instead of nasopharyngeal swabs as a clinical specimen for SARS-CoV-2 diagnostics.

Thus, in our observation regarding the clinical evaluation of 75 specimens (symptomatic and asymptomatic) that included 75 saliva samples and 75 NPS samples demonstrated comparable performance of the RNaseP. Moreover, the detection rate was higher with the proposed multiplex assay compared to the SARS-specific. Ct values for SARS-CoV-2 with the multiplex assay were comparable to N and ORF1ab genes with the SARS-specific assay. While many studies have shown that saliva samples have higher and more stable viral loads than found in NP swabs (Wyllie *et al*. 2020; Fan *et al*. 2021). Most studies detecting viral RNA in saliva have been evaluated in symptomatic or hospitalized patients, but also from asymptomatic individuals.

In summary, while alternative specimens (particularly saliva and OP/NS samples) show promise, we find that the literature to date suggests that NP swabs are indeed the gold standard in comparison to alternative specimen types (saliva, OP swab, NS). The results are heterogenous, with saliva showing lower diagnostic accuracy in some studies, while higher in others (Wyllie *et al*. 2020; Sakanashi *et al*. 2021; Fan *et al*. 2021); with one study reporting a higher concordance to nasopharyngeal samples early after symptom onset (Tan *et al*. 2021; Mestdagh *et al*. 2021; Nasiri and Dimitrova 2021; Butler-Laporte *et al*. 2021).

## Conclusion

In this study, we demonstrated the diagnostic value of saliva as an alternative matrix for SARS-CoV-2 molecular detection. Given the need for testing, the end goals are a quick, accurate and reproducible method; our findings demonstrate the potential of saliva specimens in the diagnosis of SARS-CoV-2 infection. These findings support larger-scale research on the use of saliva nucleic acid amplification testing as an alternative to nasopharyngeal swabs.

### Conflict of Interest Statement

No conflicts of interest, financial or otherwise, are declared by the author(s).

**List of Abréviations:**
ACE2: Angiotensin-Converting Enzyme 2
CoV: Coronavirus
Ct: Cycle threshold
NPS: nasopharyngeal swab
OP: *Oropharyngeal*
ORF: Open Reading Frames.
RT-qPCR: real-time quantitative reverse transcriptase-polymerase chain reaction
SARS-CoV-2: Severe Acute Respiratory Syndrome Coronavirus-2
TMPRSS2: Transmembrane Serine Protease 2.
